# Generation of Small Colony Variants in Biofilms by *Escherichia coli* Harboring a Conjugative F Plasmid

**DOI:** 10.1264/jsme2.ME16121

**Published:** 2017-03-08

**Authors:** Yosuke Tashiro, Hiroaki Eida, Satoshi Ishii, Hiroyuki Futamata, Satoshi Okabe

**Affiliations:** 1 Division of Environmental Engineering, Faculty of Engineering, Hokkaido UniversitySapporo, Hokkaido, 060–8628Japan; 2 Applied Chemistry and Biochemical Engineering Course, Department of Engineering, Graduate School of Integrated Science and Technology, Shizuoka UniversityHamamatsu, ShizuokaJapan; 3 Department of Soil, Water and Climate, University of Minnesota258 Borlaug Hall, 1991 Upper Buford Circle, St. Paul, MN 55108USA

**Keywords:** small colony variants, *Escherichia coli*, F plasmid, biofilm

## Abstract

A conjugative F plasmid induces mature biofilm formation by *Escherichia coli* by promoting F-pili-mediated cell-cell interactions and increasing the expression of biofilm-related genes. We herein demonstrated another function for the F plasmid in *E. coli* biofilms; it contributes to the emergence of genetic and phenotypic variations by spontaneous mutations. Small colony variants (SCVs) were more frequently generated in a continuous flow-cell biofilm than in the planktonic state of *E. coli* harboring the F plasmid. *E. coli* SCVs represented typical phenotypic changes such as slower growth, less biofilm formation, and greater resistance to aminoglycoside antibiotics than the parent strain. Genomic and complementation analyses indicated that the small colony phenotype was caused by the insertion of Tn*1000*, which was originally localized in the F plasmid, into the hemB gene. Furthermore, the Tn*1000* insertion was removed from *hemB* in the revertant, which showed a normal colony phenotype. This study revealed that the F plasmid has the potential to increase genetic variations not only by horizontal gene transfer via F pili, but also by site-specific recombination within a single cell.

Diversity is one of the strategies that equip organisms with the ability to protect communities from various environmental conditions. Phenotypic and genetic variations provide micro-organisms with the benefit of tolerance against environmental stresses. It has been increasingly shown that phenotypic variations occur during biofilm growth or high cell-density populations, such as the emergence of persister cells (11, 37, 39) and occurrence of colony variants (3, 4). A biofilm is a matrix-encased microbial population attached to a surface, and extracellular matrices are composed of polysaccharides, extracellular DNA, and proteins. Diverse variants have been isolated from biofilms, but not from planktonic cells under laboratory-scale conditions (3) and in clinical practice (10, 24, 34). The evolutionary rate of cells in biofilms is known to be higher than that in the planktonic state, suggesting that biofilms are a favorable environment for generating variations.

Small colony variants (SCVs) are naturally occurring mutants with smaller colony sizes and increased resistance to antibiotics, such as aminoglycosides, typically due to a lower growth rate than that of normal cells (25). The phenomenon of SCVs has been reported for a wide range of species including *Staphylococcus* spp., *Pseudomonas* spp., *Burkholderia* spp., and diverse Enterobacteriaceae (6, 12, 15, 25). Previous studies revealed common characteristics: *i.e.*, SCVs were isolated under selective growth conditions in the presence of antibiotics. SCVs may also function as auxotrophs for hemin, menaquinone, or thiamine (1, 31, 43). Revertants with normal colony sizes and antibiotic tolerance have been suggested to emerge from SCVs (13, 14). In some cases, the insertion and deletion of transposable genetic elements have been responsible for the appearance of SCVs and their revertants (14). However, the mechanisms responsible for the emergence of SCVs and their revertants currently remain unclear. Since many SCVs exhibited greater antibiotic resistance and are associated with chronic infections by pathogens (10, 22, 32), their eradication is clinically important.

The F (fertility) plasmid is a mobilizable conjugative plasmid that enables horizontal gene transfer in microbial populations. It has been suggested to promote the formation of mature mushroom-like biofilms for *E. coli* K12 (9, 18, 27); however, K12 without the F plasmid is not capable of forming robust biofilms (27). The F plasmid not only synthesizes F-pili and supports cell-cell interactions, it also induces the expression of colanic acid and curli amyloid fibers, which comprise part of the *E. coli* extracellular matrix (16). Furthermore, a F-pili-specific phage was shown to inhibit the development of biofilm formation in *E. coli* carrying a natural F plasmid (19). Thus, the F plasmid plays roles in horizontal gene transfer by conjugation and in the formation of three-dimensional mature biofilms.

Although the importance of the F plasmid in biofilm formation has been discussed in detail, limited information is available on the relationship between the F plasmid and phenotypic variations in biofilms. We herein demonstrated that SCVs appeared at higher frequencies in biofilms than in the planktonic state for *E. coli* K12 strain MG1655 harboring a natural IncF F plasmid. *E. coli* SCV possessed distinct features, including high resistance to aminoglycoside antibiotics and defective biofilm formation. Genomic and complementation analyses revealed that the appearance of SCVs was caused by the insertion of the transposable element Tn*1000*, originally localized in the F plasmid, into the *hemB* gene on the chromosome. Our results suggest that the F plasmid plays important roles in the generation of variants in biofilms as well as in the formation of mature biofilms.

## Materials and Methods

### Bacterial strain, plasmids, and growth conditions

*E. coli* K12 strain MG1655 (λ^−^ *ilvG rfb-50 rph-1*) and strain MG1655 harboring a natural IncF F plasmid (16) were used in the present study. A plasmid pCA24N-hemB was obtained from the Genome Analysis Project in Japan. Bacterial cells were grown in Difco Antibiotic Medium 3 (AM3) (BD, Franklin Lakes, NJ, USA) at 37°C. The minimal inhibition concentrations (MIC) of antibiotics were assessed with AM3 containing 1.5% agar.

### Isolation of SCVs

SCVs were isolated from three different growth conditions: planktonic cultures, microtiter plate biofilms, and continuous flow-cell biofilms. Under planktonic culture conditions, bacterial cells were grown in 30 mL AM3 medium in a 50-mL tube with shaking. After a 24-h incubation, cells were pelleted by centrifugation and resuspended in 30 mL fresh medium. This was repeated ten times; therefore, cells were grown for 240 h in total.

Under microtiter plate biofilm conditions, bacterial cells were statically grown on U-type 96-well polystyrene microtiter plates (TPP, Trasadingen, Switzerland). Culture medium was replaced with fresh AM3 medium every 24 h ten times; therefore, bacterial cells that attached to wall surfaces (=biofilms) were grown for 240 h. The attached bacterial cells were detached by ultrasonication.

Under continuous flow cell biofilm conditions, bacterial cells were grown in 1/2 strength AM3 medium using a convertible flow cell chamber (model CFCAS0004, Stovall, Greensboro, NC, USA). In order to facilitate bacterial cell attachment to the glass surface of the chamber, the flow cell was initially operated in the batch mode for 2 h, and, thereafter, medium flow was initiated at a constant rate of 0.7 mL min^−1^ using a peristaltic pump. After a 240-h incubation, planktonic cells in the flow cell were discarded and only attached bacterial cells were collected and grown on agar plates.

### Quantification of biofilm formation

Biofilms were quantified according to previously reported methods (17, 23) with some modifications. Briefly, cells were grown in 100 μL of AM3 medium on U-type 96-well polystyrene microtiter plates at 37°C. Planktonic cells were removed by washing with 0.85% NaCl using a microplate washer (ImmunoWash 1575; Bio-Rad, Hercules, CA, USA), and the remaining biofilm was stained with 0.1% crystal violet. After washing the stained biofilm with 0.85% NaCl using the microplate washer, biofilm-bound crystal violet was eluted in acetone:ethanol solution (1:4 by volume) and absorbance at 570 nm was measured using a microplate reader (ARVO 1420 multilabel counter; Perkin Elmer, Waltham, MA, USA).

### Assessment of MICs of antibiotics

Comparative MIC assessments were performed with AM3 medium agar containing diluted antibiotics. Cells were grown in AM3 medium until they reached the stationary phase, and were then diluted to an optical density at 600 nm (OD_600_) of 0.1. Five microliters of diluted cells were spotted onto AM3 medium agar with antibiotics and grown at 37°C for 24 h. In the case of *E. coli* cells harboring pCA24N, 25 μg mL^−1^ chloramphenicol was added to AM3 medium agar.

### Repetitive extragenic palindromic (rep)-PCR

Rep-PCR was conducted to analyze the sequence identity of strains with the BOX A1R primer (5′-CTACGGCAAGGCGAC GCTGACG-3′). A PCR mixture was prepared according to a previously reported method (26). The conditions used for PCR were as follows: 2 min of initial denaturation at 95°C, followed by 30 cycles consisting of 94°C for 3 s, 92°C for 30 s, 50°C for 1 min, and 65°C for 8 min. Electrophoresis was conducted at 4°C overnight at 70 V with constant buffer recirculation.

### Genome analyses

Genomic DNA was extracted from bacterial pellets using a PowerSoil DNA Isolation Kit (MO BIO, Carlsbad, CA, USA) according to the manufacturer’s instructions. Genome sequencing was conducted using paired-end sequencing on the genome analyzer Hiseq (Illumina, San Diego, CA, USA) by Hokkaido System Science (Sapporo, Japan). Sequence reads were assembled using Velvet ver. 1.2.8 (44). The highest available assembly *k*-mer parameter (hash length) of 95 was used for all Velvet assembles. Reads were aligned against the *E. coli* K12 strain MG1655 genome (GenBank accession no. NC_000913) by using Mauve-Multiple Genome Alignment Software (7) and mutation sites were detected.

The sequences of the 16S rRNA gene, *hemB*, and *menC* were confirmed by conventional Sanger sequencing. Briefly, each fragment was amplified using PrimeStar GXL DNA polymerase (Takara, Otsu, Japan) and the primers 27F (5′-AGAGTTTGATCMTGGCT CAG-3′) and 1492R (5′-TACGGYTACCTTGTTACGACTT-3′) for the 16S rRNA gene, hemB-F (5′-AGACAACACTTAGCCTTAA CGA-3′) and hemB-R (5′-CTGACATAACGATCATTTCTGG-3′) for *hemB*, and menC-F (5′-GTATACCGCTGGCAGATCCC-3′) and menC-R (5′-CCTCCAGCAACAGATTCACC-3′) for *menC*, respectively. The conditions used for PCR were as follows: initial denaturation at 98°C for 4 min, and 30 cycles at 98°C for 10 s, 55°C for 15 s, 68°C for 1 min, and 72°C for 3 min. Purified PCR fragments were sequenced using a 3730 *xl* sequencer (Applied Biosystems) by the Dragon Genomics Center (Takara).

## Results

### Emergence of SCVs

**Fig. 1:**
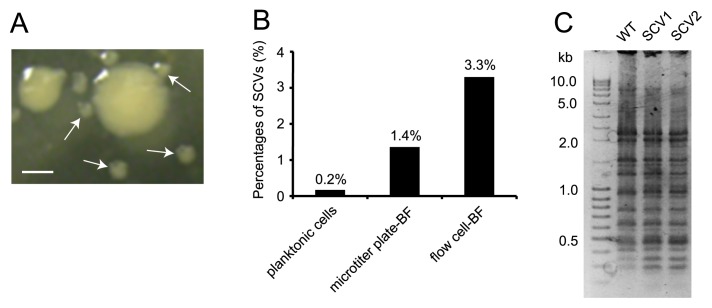
SCVs emerged from *E. coli* harboring the F plasmid. (A) Normal colony and SCVs (white arrows) on agar produced by a 5-d-old biofilm in the flow cell. Bar=1 μm. (B) The rates of SCV emergence under planktonic growth and biofilm growth conditions such as the 96-well plate assay and flow cell assay after a 5-d incubation. (C) Band patterns of rep-PCR results of WT and two SCV cells isolated independently.

In the course of the experiment, we observed the emergence of SCVs from the *E. coli* MG1655 harboring the F plasmid bacterial culture when bacteria were plated on standard agar (Fig. 1A). SCVs appeared in 0.2% (6/2,935 colonies), 1.4% (24/1,686 colonies), and 3.3% (118/3,587 colonies) of the planktonic culture, microtiter plate-based biofilm, and flow cell-based biofilm, respectively (Fig. 1B). Higher frequencies of SCV emergence under biofilm conditions suggested that biofilms induced genetic diversity, which was consistent with previous findings obtained from *P. aeruginosa* (3, 41). Although the emergence of wrinkly or large colony variants, in addition to SCVs, was previously reported in studies on *P. aeruginosa* (3, 41), no other colony morphological change other than SCVs was observed in *E. coli* MG1655. Moreover, no SCV was observed in the planktonic culture or biofilm cultures when *E. coli* MG1655, which does not harbor the F plasmid, was used, suggesting that the F plasmid is responsible for the emergence of SCV.

The 16S rRNA gene sequencing analysis confirmed that normal colony cells (WT) and SCVs had 100% identical sequences to that of *E. coli* MG1655 (GenBank accession no. NC_000913). Rep-PCR was performed to further corroborate whether the colony types of WT and SCVs are clonally related. The results obtained showed that the same band patterns were observed between WT and SCVs (Fig. 1C), indicating that SCVs were derived from *E. coli* K12 strain MG1655.

**Fig. 2:**
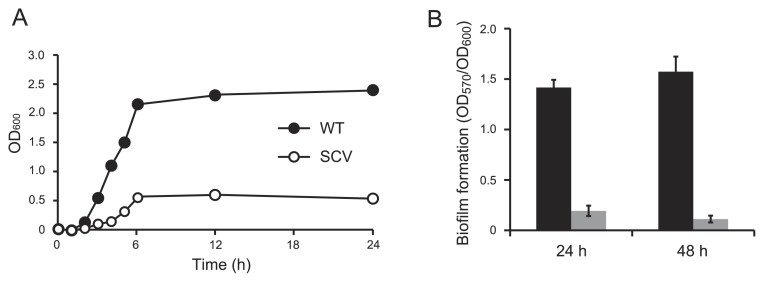
Growth curves and biofilm formation in AM3 medium at 37°C. (A) Growth curves of WT and SCV. (B) Biofilm formation by WT (black) and SCV (gray) on microtiter plates after 24-h and 48-h incubations. The amounts of biofilms were normalized by cell densities (OD_600_). Data are the average of at least six replicate wells and the standard deviations are shown.

In order to characterize SCVs, the growth and biofilm formation potentials of WT and SCVs were investigated. The growth of SCVs appeared to be markedly less than that of WT under shaking conditions (Fig. 2A). The biofilm formation potential of SCVs on microtiter plates was lower than that of WT (Fig. 2B), although SCVs were derived from biofilms at a high frequency. Thus, SCVs showed a decreased growth rate and biofilm formation.

### SCVs are resistant to aminoglycosides

In order to investigate the relationship between morphological changes and antibiotic resistance, the MICs of WT and SCVs were evaluated (Table 1). SCVs showed higher levels of resistance against aminoglycoside antibiotics including kanamycin, gentamicin, amikacin, neomycin, and streptomycin, than WT. On the other hand, WT and SCVs showed similar resistance levels to non-aminoglycoside antibiotics such as ampicillin, tetracycline, chloramphenicol, and ofloxacin. Thus, resistance to aminoglycoside antibiotics markedly differed between WT and SCVs.

### Chromosomal mutations in SCVs

In order to identify mutation sites in SCVs, genome analyses were performed using the Illumina HiSeq 2000 sequencer. A total of 40,920,072 and 41,573,080 high quality sequence read pairs of 2×101 nt lengths were obtained for WT and SCV, respectively, which corresponded to >880× coverage. Based on *de novo* assembly, we obtained 162 and 164 contigs with N_50_ of 126,004 bp and 132,634 bp for the genomes of WT and SCV, respectively.

**Fig. 3:**
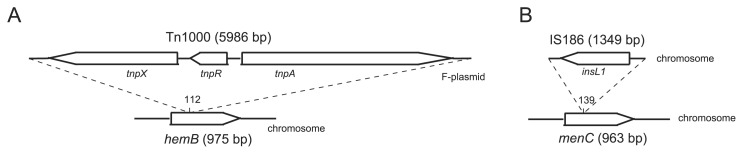
Two mutation sites in SCV. (A) The insertion of Tn*1000* localized in the F plasmid into chromosomal *hemB*. (B) The insertion of IS*186* on the chromosome into *menC*.

We identified three differences between WT and SCV chromosomes. One difference was the insertion of the transposon Tn*1000*, constituting 5,986 bp, from the F plasmid into the chromosomal *hemB* at the position of 112 bp (Fig. 3A). HemB is a 5-aminolevulinate dehydratase that is required for hemin synthesis (20). Previous studies reported that the inactivation of the heme biosynthetic pathway is one possible cause for the appearance of SCVs, and our results are consistent with previous findings (14, 30). The second mutation was the insertion of IS*186*, constituting 1349 bp, from the K12 chromosome into chromosomal *menC* at the position of 139 bp, which is O-succinylbenzoyl-CoA synthase and is required for menaquinone (vitamin K2) synthesis (33). Since previous studies indicated that some menaquinone-deficient mutants had the small colony phenotype (31), the mutation of *menC* may also be associated with the SCV phenotype. The insertions of Tn*1000* and IS*186* into *hemB* and *menC*, respectively, were also confirmed by Sanger sequencing. Thirdly, an approximately 56-kb region starting at *gatC* and ending at *yohP* in SCVs was transferred to a different position from that in WT; however, no mutations were observed in each gene localized in the transferred region.

### Association of *hemB* with SCV phenotypes

**Fig. 4:**
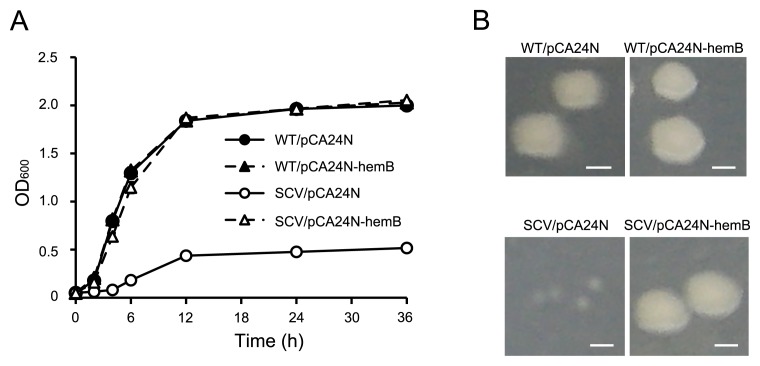
Complementation of *hemB* recovers growth and colony phenotypes of SCV. (A) Growth curves of WT and SCV harboring the control plasmid pCA24N or complementation plasmid pCA24N-hemB in AM3 medium containing chloramphenicol at 37°C. (B) Colony phenotypes of WT/pCA24N, WT/pCA24N-hemB, SCV/pCA24N, and SCV/pCA24N-hemB. Bar=1 μm.

We next investigated whether SCV phenotypes depend on defective *hemB* and/or *menC*. Since a previous study demonstrated that aminoglycoside resistance increased in a hemindeficient mutant (2), we hypothesized that a mutation in *hemB*, but not in *menC* is responsible for the morphological change from WT to SCV. In order to corroborate this hypothesis, *hemB* was complemented in SCV using the expression vector pCA24N containing *hemB*. The growth of SCV was recovered by the expression of *hemB* (Fig. 4A). Although pCA24N has the *lac* promoter and IPTG is needed to moderately express the gene located downstream of the *lac* promoter, SCV/pCA24N-hemB showed similar growth to WT/pCA24N without IPTG, suggesting that the weak of *hemB* expression is to restore normal growth in SCVs. When plasmid-cured cells, in which the plasmid was removed, were obtained from strain SCV/pCA24N-hemB by culturing without chloramphenicol, the growth rate became slower again, similar to SCV (data not shown). We also confirmed that SCV/pCA24N-hemB showed the WT-like normal colony type (Fig. 4B). Moreover, MIC results revealed that the expression of *hemB* recovered aminoglycoside sensitivity in SCV (Table 2). These results indicate that SCV characteristics such as slow growth, the small colony phenotype, and aminoglycoside resistance are attributed to a mutation in *hemB*.

### Characterization of the SCV revertant

**Fig. 5:**
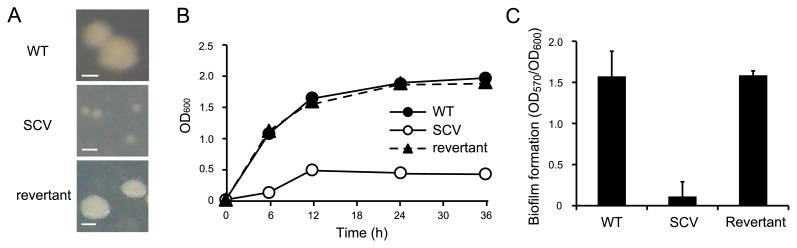
The revertant showed WT-like phenotypes. (A) Colony phenotypes of the WT, SCV, and revertant. (B) Growth curves of the WT, SCV, and revertant in AM3 medium at 37°C. (C) Biofilm formation by the WT, SCV, and revertant on microtiter plates after a 48-h incubation in AM3 medium at 37°C. The amounts of biofilms were normalized by cell densities (OD_600_). Data are the average of at least six replicate wells and standard deviations are shown.

We then investigated the effects of the addition of hemin on SCV growth. A previous study showed that the SCVs of *E. coli* were not permeable to hemin (28), and our results are consistent with these findings: the addition of hemin to the LB culture did not recover the growth of *hemB*-deficient SCVs (data not shown). However, when grown with hemin, we obtained revertants that showed the normal colony pheno-type (Fig. 5A). Furthermore, the growth of and biofilm formation by the revertants were similar to those of WT (Fig. 5B and C). Similar antibiotic resistance was also observed between the revertant and WT (Table 1).

**Fig. 6:**
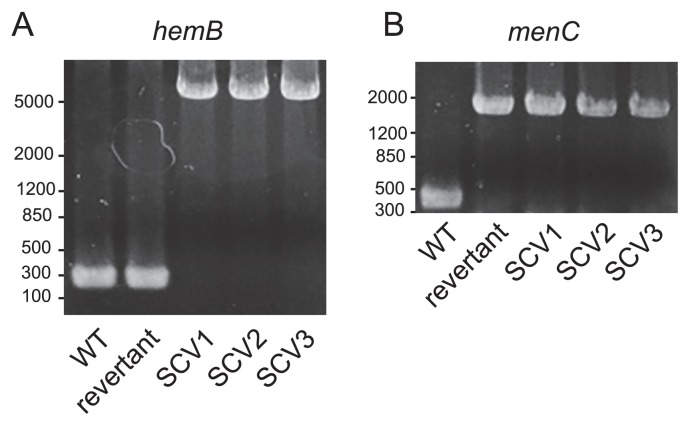
PCR results by amplifying *hemB* (A) and *menC* (B) regions in chromosomes of the WT, revertant, and three SCVs.

In order to identify the genetic changes that induced the phenotypes of the revertant, we analyzed the sequences of the *hemB* and *menC* regions in the chromosomes of WT, SCV, and the revertant. The insertion of Tn*1000* in *hemB*, which was observed in SCVs, was not detected in the revertant (Fig. 6A). In addition, the *hemB* sequence of the revertant was identical to that of WT. In contrast, *menC* of the revertant was the same as that of SCV (Fig. 6B). Collectively, these results indicated that the emergence of the revertant was at least associated with the deletion of Tn*1000* from *hemB*.

## Discussion

Although F plasmids, which are widespread in *E. coli* and other enterobacteria, are important for mature biofilm formation (9, 16, 27), the influence of the F plasmid on the emergence of variants in biofilms remains largely unknown. In the present study performed using *E. coli* MG1655 harboring a natural IncF F plasmid, we found that i) SCVs more frequently appeared in biofilms than under planktonic growth conditions; ii) the growth of and biofilm formation by SCVs were lower than those of the parent strain; iii) SCVs showed greater aminoglycoside antibiotic resistance than the parent strain, and iv) the small colony phenotype was caused by the insertion of Tn*1000*, which was originally localized in the F plasmid, into the *hemB* gene. This is the first study to show that the F plasmid yields phenotypic variations at the single cell level in *E. coli*.

Diverse genetic or phenotypic variants have been generated during biofilm growth in a wide range of bacterial species. The emergence of *P. aeruginosa* SCVs has frequently been reported (3, 36, 40). One study demonstrated that endogenous oxidative stress, which increases under biofilm conditions, caused double-stranded DNA breaks in some biofilm cells, and genetic variants were generated when breaks were repaired (4).

Prophage excitation has so far been reported as the transposition of DNA elements in the biofilms of *E. coli* and *P. aeruginosa* (38, 40). Prophage genes are more strongly induced in biofilms than in the planktonic state in *E. coli*, *P. aeruginosa*, and *Bacillus subtilis* (8, 35, 42). Prophage excision increases the generation of variants including SCVs in *P. aeruginosa* (40); however, regulatory mechanisms for prophage excision in this bacterium currently remain unclear. In *E. coli*, the global regulator, Hha induces the excitation of prophages by enhancing their transcription (38). Since the frequency of SCV emergence was higher in biofilms than in the planktonic state, Tn*1000* also appears to be more transposable in biofilms than in the planktonic state. A clearer understanding of the behavior of transposons in biofilms will result in the mechanisms underlying variant generation in biofilms being elucidated, and, thus, warrants further study.

The results of the whole genome analysis showed that there were two genetic mutation sites in *E. coli* SCVs: the insertion of Tn*1000* from the F plasmid into *hemB* and the insertion of IS*186* from the chromosome into *menC*. When *hemB* was expressed by the expression vector pCA24N-hemB in SCVs, unique SCV characteristics including growth, antibiotic resistance, and colony morphology were complemented, indicating that the mutation of *hemB* is the primary factor to induce the emergence of SCVs. Similar to our study, previous genetic analyses also showed that the deletion of *hemB* was associated with the SCV phenotype (14, 20, 30). We found that the insertion in *hemB* was removed in the revertant, whereas the insertion in *menC* remained. These results also reinforce the emergence of SCVs being due to the mutation of *hemB*. The *hemB* gene is considered to be a hot spot for spontaneous mutations in *E. coli* K12 because more than 80% of 150 independently isolated respiratory-deficient mutants were found to have mutations in *hemB* (13). In a previous study, IS2 was inserted into the *hemB* gene in SCV derived from *E. coli* K12 (14). We analyzed 10 SCV colonies isolated from independent cultures in our experiments; however, all SCVs had the insertion of Tn*1000* in *hemB* (data not shown), suggesting that *hemB* is more sensitive to the insertion of Tn*1000* than other transposable elements in the presence of the F plasmid. We focused on SCVs in the present study; however, it is important to note that genetic variants other than SCVs may have been generated in *E. coli* biofilms, and SCVs may only account for a very small percentage of the large amount of variations occurring in biofilms.

SCVs isolated in this study showed decreased biofilm formation, although SCVs emerged at a high frequency under biofilm conditions. In earlier studies performed with *P. aeruginosa*, two types of SCVs were isolated: one type lacked the ability to form biofilms and easily detached from biofilms (3), while the another type showed an enhanced ability to form biofilms (15, 36, 40). Thus, SCVs isolated under different conditions have distinct characteristics even when they originate from the same species. Hyper-biofilm-forming *P. aeruginosa* SCVs are capable of overproducing extracellular polysaccharides (15, 36), while *E. coli* SCVs showed decreased biofilm formation (Fig. 2B). A role for SCVs in biofilm development has not yet been established in *E. coli*, with the exception of antibiotic resistance; however, further investigations will contribute to our understanding of the importance of SCV emergence in biofilm development.

Resistance to aminoglycoside antibiotics in SCVs has been reported in several bacterial species including *E. coli* and *S. mutans* (13, 25). In any of these bacterial species, the appearance of SCVs has been associated with the inactivation of the heme biosynthetic pathway. Hemin has been suggested to be associated with aminoglycoside susceptibility, rapid growth, and large colonies (25). It is also required for the synthesis of cytochrome, which directly activates the synthesis of F_0_F_1_-ATPase. A decrease in F_0_F_1_-ATPase causes a membrane potential (ΔΨ) and ΔΨ is known to facilitate aminoglycoside uptake (5, 21). Aminoglycoside resistance in SCVs has been attributed to a specific defect in electron transport. A decrease in ATP in the cytoplasm may also be caused by a defect in F_0_F_1_-ATPase. A decrease in ATP may repress cell-wall biosynthesis and protein synthesis, causing slower growth and smaller colony sizes of SCVs than those of WT. On the other hand, a *hemB* mutation was not reported in *P. aeruginosa* SCVs, but also shows aminoglycoside resistance (41). Since the overexpression of efflux pumps may increase aminoglycoside resistance in *P. aeruginosa* SCVs, the mechanisms underlying the aminoglycoside resistance of SCVs are not common among bacteria.

A recent study indicated that the mutation of *lipA*, which is the lipoic acid synthase gene, leads to the SCV phenotype and slow growth in *E. coli* BW25113 (29). This mutant showed high resistance to several stresses including acid, hydrogen peroxide, heat, and osmotic stresses. Since the expression of genes involved in glycolysis, the TCA cycle, and electron transport are repressed in the *lipA* mutant, a low cellular ATP level leads to slow growth and high resistance. Thus, our results are consistent with previous findings showing that a defect in an auxotrophic molecule is one of the reasons for the appearance of SCVs with higher resistance to several stresses.

In conclusion, the results of the present study indicate that a biofilm-promoting F plasmid contributes to the emergence of genetic variants by transferring transposon Tn*1000* to chromosomal *hemB* in *E. coli*. Thus, the F plasmid has the potential to increase genetic variations not only by horizontal gene transfer using F pili, but also by site-specific recombination within a single cell. The F plasmid is essential for mature biofilm formation in which genetic variants are concomitantly generated. Bacteria present in biofilms have the advantage over planktonic cells of tolerance against environmental perturbations. This study provides new insights into the generation of variants in microbial biofilm communities.

## Figures and Tables

**Table 1:** MIC of *E. coli* MG1655.

Antibiotics	MIC (μg mL^−1^)		

	WT	SCV	Revertant
Ampicillin	8	4	4
Chloramphenicol	8	4	8
Tetracycline	2	1	2
Ofloxacin	0.25	0.13	0.25
Kanamycin	16	64	16
Gentamicin	8	64	8
Amikacin	8	64	8
Neomycin	4	64	4
Streptomycin	16	64	16

**Table 2:** MIC of *E. coli* MG1655 harboring pCA24N-series.

Antibiotics	MIC (μg mL^−1^)			

	WT/pCA24N	SCV/pCA24N	SCV/pCA24N-hemB	SCV/pCA24N-hemB
Ampicillin	8	8	8	8
Tetracycline	2	2	1	1
Ofloxacin	0.25	0.25	0.125	0.25
Kanamycin	16	16	64	16
Gentamicin	8	8	64	8
Amikacin	8	8	64	8
Neomycin	8	8	64	8
Streptomycin	16	16	64	16
